# De novo engineering riboflavin production *Bacillus subtilis* by overexpressing the downstream genes in the purine biosynthesis pathway

**DOI:** 10.1186/s12934-024-02426-w

**Published:** 2024-05-31

**Authors:** Chuan Liu, Miaomiao Xia, Huan Fang, Fan Xu, Sijia Wang, Dawei Zhang

**Affiliations:** 1 Tianjin Institute of Industrial Biotechnology, Chinese Academy of Sciences, Tianjin, 300308 China; 2 Key Laboratory of Engineering Biology for Low-Carbon Manufacturing, Tianjin Institute of Industrial Biotechnology, Chinese Academy of Sciences, 32 West 7th Avenue, Tianjin Airport Economic Area, Tianjin, 300308 China; 3 University of Chinese Academy of Sciences, Beijing, 100049 China; 4 School of Chemical Engineering, Hebei University of Technology, Tianjin, 300131 China; 5 School of Biological Engineering, Dalian Polytechnic University, Dalian, 116034 China

## Abstract

**Background:**

*Bacillus subtilis* is widely used in industrial-scale riboflavin production. Previous studies have shown that targeted mutagenesis of the ribulose 5-phosphate 3-epimerase in *B. subtilis* can significantly enhance riboflavin production. This modification also leads to an increase in purine intermediate concentrations in the medium. Interestingly, *B. subtilis* exhibits remarkable efficiency in purine nucleoside synthesis, often exceeding riboflavin yields. These observations highlight the importance of the conversion steps from inosine-5’-monophosphate (IMP) to 2,5-diamino-6-ribosylamino-4(3 H)-pyrimidinone-5’-phosphate (DARPP) in riboflavin production by *B. subtilis.* However, research elucidating the specific impact of these reactions on riboflavin production remains limited.

**Result:**

We expressed the genes encoding enzymes involved in these reactions (*guaB*, *guaA*, *gmk*, *ndk*, *ribA*) using a synthetic operon. Introduction of the plasmid carrying this synthetic operon led to a 3.09-fold increase in riboflavin production compared to the control strain. Exclusion of *gmk* from the synthetic operon resulted in a 36% decrease in riboflavin production, which was further reduced when *guaB* and *guaA* were not co-expressed. By integrating the synthetic operon into the genome and employing additional engineering strategies, we achieved riboflavin production levels of 2702 mg/L. Medium optimization further increased production to 3477 mg/L, with a yield of 0.0869 g riboflavin per g of sucrose.

**Conclusion:**

The conversion steps from IMP to DARPP play a critical role in riboflavin production by *B. subtilis*. Our overexpression strategies have demonstrated their effectiveness in overcoming these limiting factors and enhancing riboflavin production.

**Supplementary Information:**

The online version contains supplementary material available at 10.1186/s12934-024-02426-w.

## Background

Riboflavin, an indispensable vitamin, has wide applications in the food, feed, and pharmaceutical industries [1–3]. The two active forms of riboflavin, flavin adenine dinucleotide (FAD) and flavin mononucleotide (FMN), are crucial for diverse cellular processes, including carbohydrate, lipid, and protein metabolism [4–6]. However, humans and other animals lack the ability to *de novo* synthesize riboflavin, necessitating a dietary intake. In contrast, microorganisms, including specific mutants of *Bacillus subtilis* [7], possess *de novo* riboflavin biosynthesis pathways and can excrete riboflavin into the environment [8, 9].

In recent decades, the commercial production of riboflavin through fermentation has significantly flourished, surpassing chemical synthesis. This trend is partly due to the advantages offered by *B. subtilis*, which is characterized by rapid growth, minimal nutritional requirements, and facile product extraction from fermentation broth [10–12]. Furthermore, advancements in genetic engineering have enabled *B. subtilis* to emerge as a highly competitive riboflavin producer, driving industrial-scale production efforts. Recently, the riboflavin market has been valued at USD 4.23 billion and is anticipated to continue growing [13]. This growth is attributed to the versatile uses of riboflavin, which serves as a valuable component in various products ranging from feed additives to cardiac medications and anticancer drugs.

In riboflavin biosynthesis, guanosine triphosphate (GTP) and ribulose-5-phosphate (Ru5P) serve as the two committed precursors. Our previous investigation identified a mutation in ribulose 5-phosphate-3-epimerase (RPE) that contributes to riboflavin overproduction in a *B. subtilis* strain with deregulated *rib* operon [14]. This mutation reduces the carbon flux to the Embden-Meyerhof-Parnas (EMP) pathway, thereby enhancing the precursor supply for riboflavin production, an effect akin to that observed in certain transketolase-deficient *B. subtilis* strains [15]. The augmented supply of ribose precursor results in the accumulation of purine metabolites in the fermentation media [14].

Notably, the purine *de novo* biosynthesis pathway encompasses steps beyond the conversion of Ru5P and GTP to riboflavin. The pathway is governed by complex feedback inhibition mechanisms. The transcriptional regulation of the purine operon is mediated by the PurR regulator [16]. Furthermore, upon binding to guanine, the purine riboswitch forms a terminal hairpin loop, leading to the initial termination of purine operon transcription [17]. Intermediates of the purine *de novo* biosynthesis pathway also regulate enzyme activities [18]. Several strategies aimed at enhancing riboflavin production through purine metabolism engineering have been investigated for strain development. Shi et al. achieved a 3-fold increase in riboflavin production (826.5 mg/L) by enhancing the purine *de novo* synthesis pathway through a series of deregulatory approaches [19]. Sun et al. pinpointed crucial genes within the purine salvage and degradation pathway that impact riboflavin production, paving the way for more precise manipulation of purine metabolic networks [20]. Nonetheless, data on intermediate concentrations and potential regulatory mechanisms from these studies implicate the necessity of expressing downstream genes in the GTP biosynthesis pathway. Based on this observation, we hypothesize that the regulation of the pathway by intermediate products can be alleviated through the overexpression of these genes alone.

To test this hypothesis, riboflavin production strain is constructed through the expression of five genes ranging from inosine-5’-monophosphate (IMP) to 2,5-diamino-6-ribosylamino-4(3 H)-pyrimidinone-5’-phosphate (DARPP), and the production of riboflavin was subsequently tested via flask fermentation. The expression of either all or a subset of these genes resulted in enhanced riboflavin production to varying extents. The most effective engineering approach to enhancing riboflavin production proved to be the overexpression of all these genes within a synthetic operon. These results suggest that a set of genes acting as bottlenecks have been identified in the parent strain. Through additional genetic engineering efforts and optimization of the fermentation medium, the final strain achieved a riboflavin titer of up to 3477 mg/L.

## Results

### Construction of the plasmid pEX5 for overexpressing the genes involved in the conversion of IMP to DARPP

**Fig. 1:**
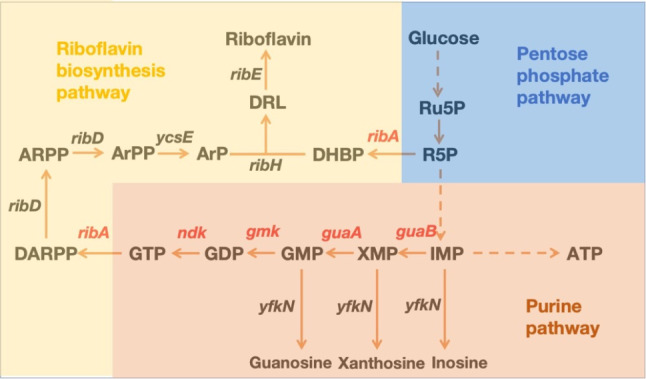
This overview provides a comprehensive perspective on the genes involved in the downstream segments of the pentose phosphate pathway (blue), the purine pathway (orange), the riboflavin biosynthesis pathway (yellow), and their interconnected pathways within the framework of *B. subtilis*. Within this intricate biochemical network, inosine monophosphate (IMP) represents the first molecule bearing an intact purine ring, and other purine nucleotide are generated from IMP. In *B. subtilis*, a series of critical genes assume central roles in these biochemical transformations. Specifically, *guaB*, responsible for encoding IMP dehydrogenase, orchestrates the conversion of IMP into XMP. Furthermore, *guaA*, encoding GMP synthetase, governs the conversion of XMP into GMP. *gmk*, the gene responsible for guanylate kinase, is instrumental in catalyzing the transition from GMP to GDP. *ndk*, denoting nucleoside diphosphate kinase, presides over the conversion from GDP to GTP. And as the first step from purine precursor to riboflavin biosynthesis, *ribA*, encoding GTP cyclohydrolase II/3,4-dihydroxy-2-butanone 4-phosphate synthase, presides over the transformation of GTP and Ru5P into DARPP and DHBP

In *Bacillus subtilis*, the genes responsible for encoding enzymes in the conversion pathway from inosine monophosphate (IMP) to 3,4-dihydroxy-2-butanone 4-phosphate (DARPP) are *guaB*, *guaA*, *gmk*, *ndk*, and *ribA* (Fig. 1). The enzymes IMP dehydrogenase (encoded by *guaB*) and Guanosine monophosphate synthetase (GMP synthetase) (encoded by *guaA*) catalyze consecutive reactions, driving the conversion of IMP to GMP. When expressing these genes, there are two notable aspects to consider.

Firstly, unlike the purine operon formed by genes involved in IMP biosynthesis, these genes are not adjacent in the genome and can be significantly distant. Table 1 presents the upstream and downstream genes adjacent to these genes in the *B. subtilis* genome, along with their respective functions. Specifically, *guaB* is located at 15,915–17,381, *guaA* at 692,740–694,281, *gmk* at 1,641,949–1,642,563, and *ndk* at 2,381,354–2,381,803 [21]. This suggests that during evolution, the strain did not prioritize GTP accumulation but required tight coordination between intermediate synthesis and other cellular processes. Given their scattered locations and functional significance, it is necessary to construct a synthetic operon for these genes.

**Table 1:** Adjacent genes to genes in downstream genes of GTP synthesis in *B. subtilis*

Genes	Location	Upstream/Downstream genes	Function
*guaB*	15,915–17,381	*yaaC*/*dacA*	Unknow/Major D-alanyl-D-alanine carboxypeptidase
*guaA*	692,740–694,281	*yebA*/*pbuG*	Unknow/Hypoxanthine and guanine uptake
*gmk*	1,641,949–1,642,563	*ylzA*/*rpoZ*	Control of biofilm formation/Omega subunit of RNA polymerase
*ndk*	2,381,345–2,381,803	*cheR*/*hepT*	Motility, chemotaxis/Menaquinone biosynthesis

Secondly, the catalytic biases observed in this series of reactions merit attention. This direction of conversion is supported by the metabolic pathway data available on Metacyc [22]. Nevertheless, an excess of purine nucleotides is subject to degradation into nucleosides through the action of various 5’-nucleotidases in *B. subtilis* [23]. Diverting this metabolic flow toward riboflavin biosynthesis necessitates the introduction of a competitive enzyme. Guanylate kinase (encoded by *gmk*) and nucleoside-diphosphate kinase (encoded by *ndk*) are pivotal in the reactions converting GMP to GTP. Since these reactions are reversible, their overexpression may not significantly alter the metabolic flow. RibA catalyzes the initial steps of riboflavin synthesis, and these reactions are irreversible, as stated in the Metacyc database. Prior research has demonstrated that the overexpression of *ribA* exerts a substantial impact on riboflavin production [24]. Consequently, RibA emerges as a suitable “driver” for the consecutive reactions.

Based on the aforementioned theory, these genes were assembled into a synthetic operon (Supplementary Fig. 1). This operon was under the control of a strong promoter called Pr, which was created by deregulating and modifying the promoter of the *rib* operon [25]. In *B. subtilis*, RibA serves as a dual-function enzyme (GTP cyclohydrolase II and 3,4-dihydroxy 2-butanone 4-phosphate synthase) responsible for key steps in the production of riboflavin [24]. Given the significance of RibA, it was positioned at the beginning of the operon. The enzyme catalyzing the penultimate step, encoded by *ndk*, is located downstream of *ribA* in the operon. The arrangement of the other three genes within the operon follows the sequence of their respective catalyzed reactions. To prevent transcription attenuation, a PvegI constitutive promoter [26] was inserted upstream of *guaB*. The mid-copy number plasmid pHP13(spe) [14, 27] is chosen as the backbone, and the operon is ligated to it by Gibson assembly to generate pEX5.

### Effect of overexpression of downstream genes of GTP synthesis pathway and *ribA* on riboflavin production

**Fig. 2:**
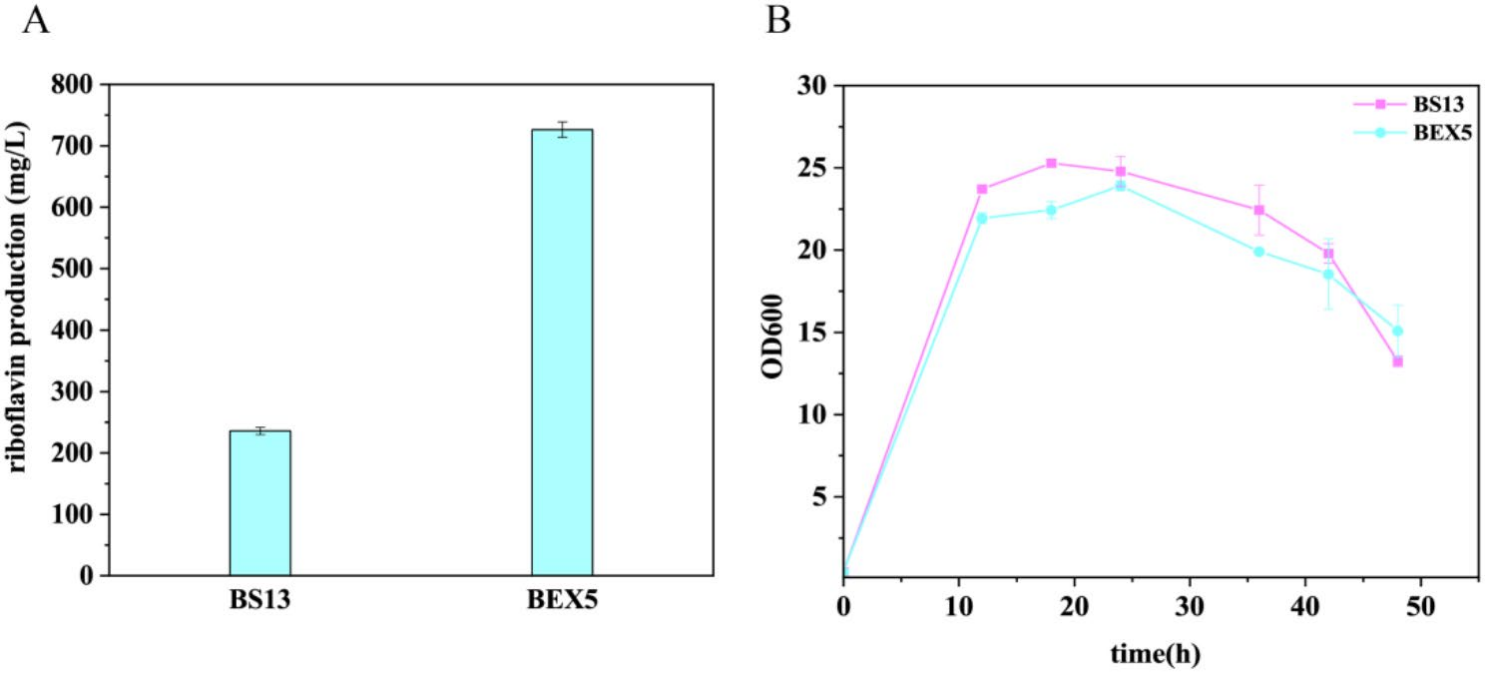
The comparative analysis of growth and riboflavin production between strains overexpressing a synthetic operon and the control strain. (**A**) Presents the riboflavin production between BEX5 (BSR overexpression pEX5) and B13 (BSR overexpression pHP13 (spe)) during flask fermentation at 48 h, with sucrose employed as the carbon source. (**B**) The growth curve of BEX5 and B13

The pEX5 is introduced into the strain BSR (Table 3) to generate BEX5. BSR is constructed on the background of *B. subtilis* 168, containing a deregulated *rib* operon and a mutation of ribulose 5-phosphate 3-epimerase (RPE), which causes a reduced RPE activity, leading to the accumulation of purine intermediates as reported in our prior investigations [14]. Flask fermentation was conducted to test BEX5 and BS13, which is BSR transformed with the pHP13(spe) vector. As shown in Fig. 2A, while BEX5’s growth was slightly impeded compared to the control strain (11.27% decrease), the introduction of the synthetic operon significantly increased riboflavin production to 726 ± 12 mg/L (a 3.09-fold increase) after 48 h (Fig. 2B). This surpassed the results of *ribA* overexpression alone and closely matched the effects of *rib* operon overexpression reported previously [14].

**Fig. 3:**
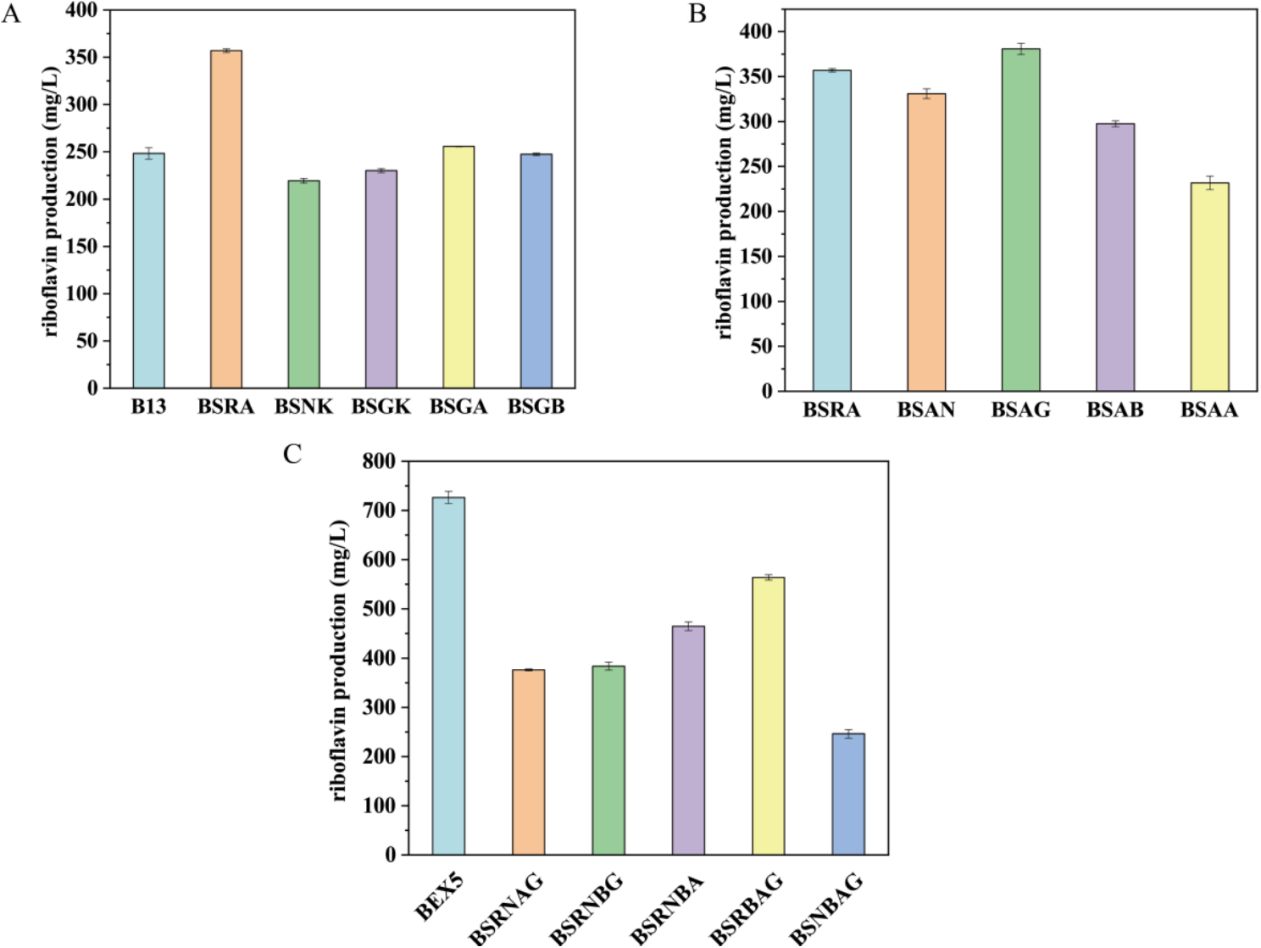
The riboflavin production performance is examined in flask fermentation at 48 h, within strains overexpressing specific genes from the synthetic operon. (**A**) Riboflavin production within strains overexpressing individual genes from the synthetic operon. (**B**) Riboflavin production when strains overexpress *ribA* in conjunction with the remaining four genes of the synthetic operon. (**C**) Riboflavin production within strains where four out of the five genes from the synthetic operon are co-overexpressed

To investigate the effect of overexpression of a single gene on riboflavin production, vectors carrying five independent genes under the control of Pr promoter were constructed and transformed into BSR to generate BSRA, BSGA, BSGB, BSGK, and BSNK. As the flask fermentation results showed, only BSRA exhibited a significant enhancement of riboflavin production compared with the control strain (Fig. 3A). The biomass of these overexpressed strains was similar to that of the control strain, except for BSGA, which was higher than the control strain (Supplementary Fig. 2).

These results indicate that overexpressing the combination of the five genes increases carbon flow towards riboflavin production, with *ribA* as the key enzyme. Further more, plasmids were constructed in which *ribA* was co-expressed with each of the other four genes individually. These plasmids were then transformed into BSR to generate BSAA, BSAB, BSAN, and BSAG. As shown in Fig. 3B, riboflavin production increased by 6.6% in the strain overexpressing the combination of *ribA* and *gmk* compared to the strain overexpressing *ribA* alone, while production decreased in other combinations. Alternatively, strains overexpressing random combinations of four of the five genes were constructed. Fermentation results demonstrated that riboflavin production decreased by 66% when *ribA* was absent compared to BSEX5. Additionally, riboflavin production decreased by 48.3%, 47.2%, 36.0%, and 22.4% in the absence of *guaB*, *guaA*, *gmk*, and *ndk*, respectively (Fig. 3C). These results demonstrate that all five genes are necessary, to varying degrees, for achieving higher riboflavin production.

In *E. coli*, the gene pairs *gmk*-*ndk* and *guaB*-*guaA* were both expressed to increase riboflavin production [28]. However, it was observed that only the *gmk*-*ndk* combination favorably impacted riboflavin production, indicating a divergence in purine metabolism between the two strains. In *B. subtilis*, inosine and IMP are the primary byproducts of purine pathways [14], whereas *E. coli* tends to accumulate xanthine when GMP synthase is knocked out [29].

### Development of a riboflavin-producing strain

**Fig. 4:**
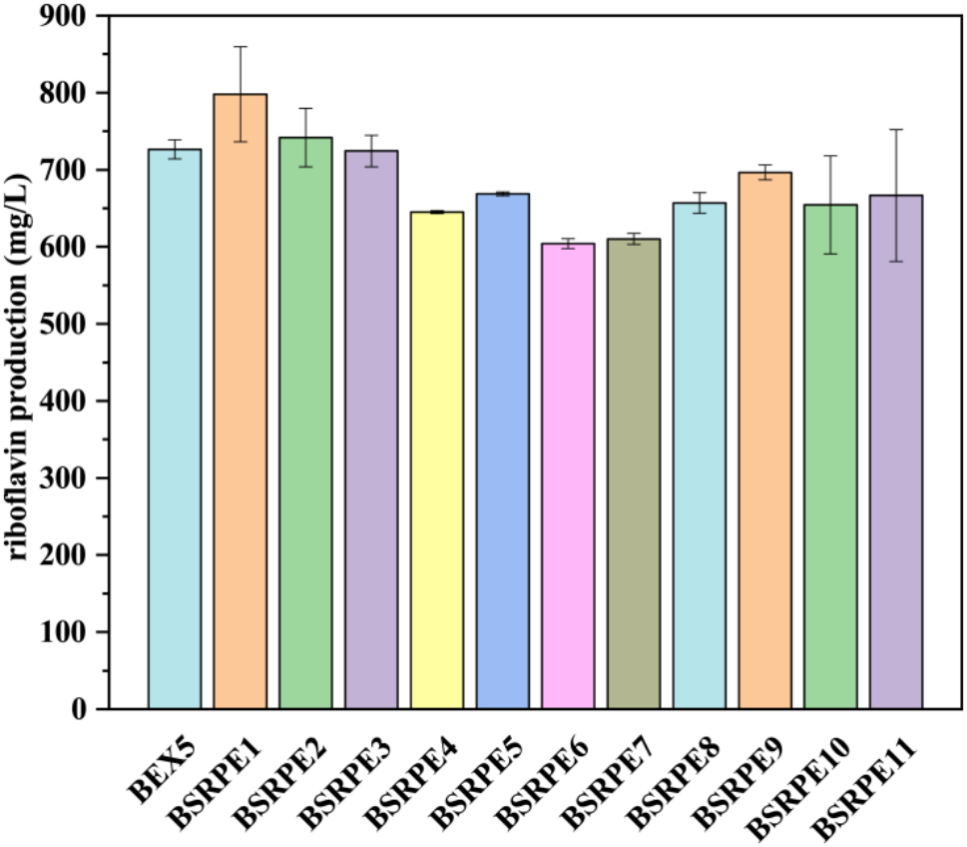
Riboflavin production was assessed in flask fermentation at 48 h, among strains with diverse genetic backgrounds, each of which overexpressed the synthetic operon. Specifically, the introduction of pEX5 was performed within deletion mutants of the purine metabolic network. Subsequently, the performance of these strains was evaluated through flask fermentation experiments

Subsequently, we combined the overexpression of the synthetic operon with other previously employed strategies in our studies to develop a more competitive riboflavin-producing strain. Initially, we generated strains by introducing pEX5 into purine metabolic network deletion mutants [20] to assess the regulatory effect on the synthetic operon. These strains were subsequently subjected to flask fermentation experiments. As shown in Fig. 4, the highest riboflavin production of 798 ± 61 mg/L was achieved in the *apt* deletion strain. Furthermore, the riboflavin production of BSRPE2 exceeded that of the parent strain. However, it was observed that gene deletions in BSRPE3-11 adversely affected strains overexpressing the synthetic operon. These findings underscore the diverse impacts of strain backgrounds on production when expressing the synthetic operon. This phenomenon is attributed to the complex regulation of the purine *de novo* synthesis pathway, which involves salvage, degradation, and interconversion pathways [20].

Subsequently, a series of genetic manipulations were performed on the background of BSR, which was derived from the wild-type strain *B. subtilis* 168 [14]. Initially, the synthetic operon was integrated into the *amyE* locus of BSR, resulting in the generation of BSRE1. Subsequently, the *apt* gene was deleted in BSRE1 to produce BSRE2, followed by the introduction of a site-directed mutation in *perR* [20] to generate BSRE3.

To further augment the supply of pentose precursors, a nonsense mutation of *tkt* [14] was introduced into BSRE3, resulting in the formation of BSRE4. Finally, plasmid pMX45, carrying an overexpressed *rib* operon, was introduced into BSRE4, yielding BSRE4/pMX45.

**Fig. 5:**
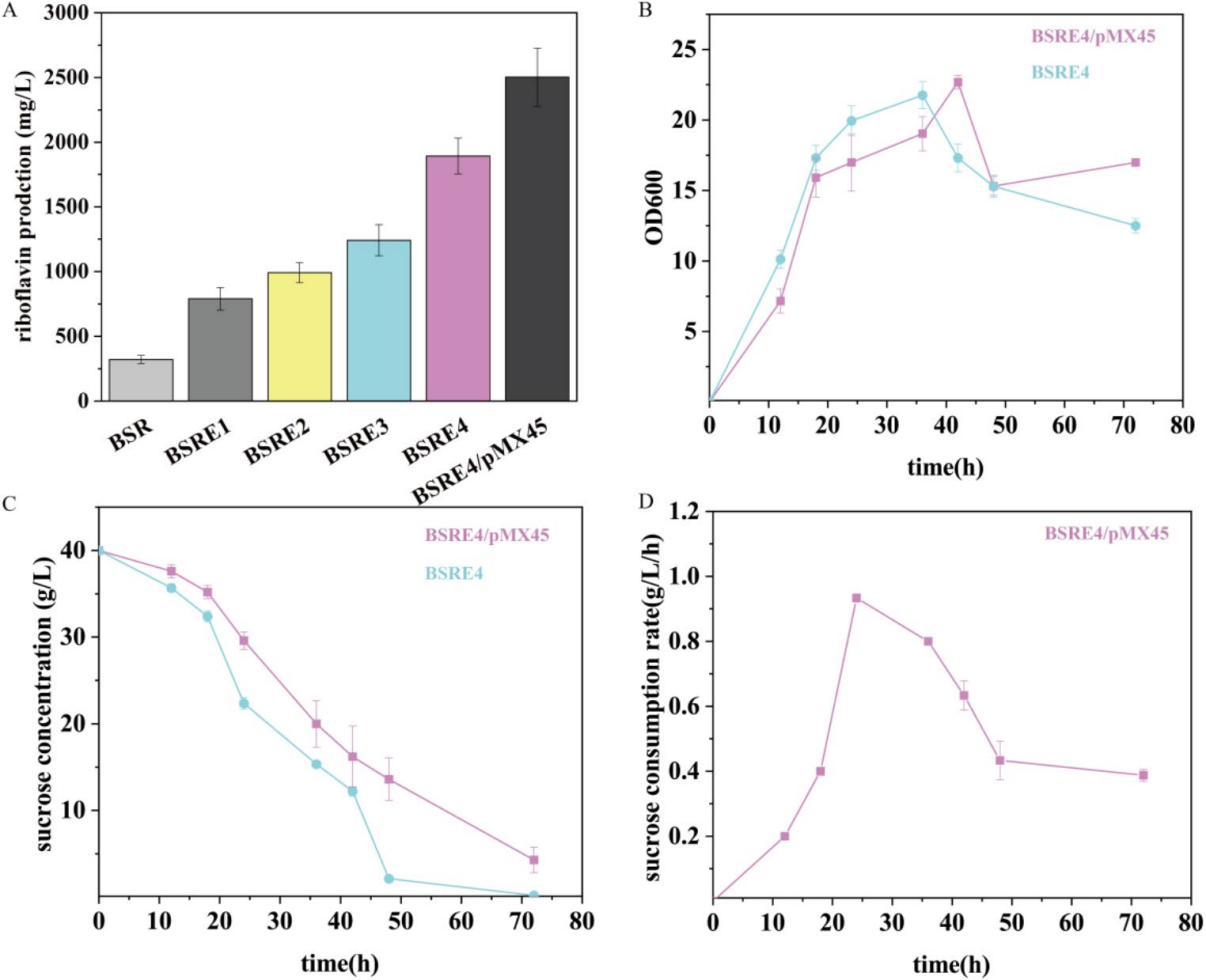
Various aspects of the genetic engineering strain, including riboflavin production, biomass, and sucrose consumption. (**A**) Riboflavin production levels in a series of strains with a genome-integrated synthetic operon, all based on the BSR background. (**B**) Comparison of the biomass between the final strain BSRE4/pMX45 and the parent strain. (**C**) The sucrose concentration within the fermentation medium for both BSRE4/pMX45 and the parent strain. (**D**) The sucrose consumption rate specifically for BSRE4/pMX45

Flask fermentation experiments were conducted to assess the riboflavin production capacity of these strains. As Fig. 5 shows, when the synthetic operon was integrated into the genome, riboflavin production reached 790 ± 87 mg/L, representing a remarkable 2.46-fold increase compared to the parent strain. Notably, the production further surged to 991 ± 78 mg/L following the knockout of the *apt* gene. PerR, a global regulator with influence over purine metabolism [30], was strategically targeted with a site-directed mutation in previous studies. This mutation suppressed the dNTP synthesis pathway, redirecting carbon flow towards GTP/riboflavin production and leading to a notable 25% increase in riboflavin production. Consequently, in BSRE4, riboflavin production soared to 2012 ± 139 mg/L, even though the biomass of BSRE4 experienced a 32% reduction compared to BSRE3 (Supplementary Fig. 3). This suggests a significant redistribution of carbon flux between growth and precursor production. The production of riboflavin in BSRE4/pMX45 reached an impressive 2702 ± 224 mg/L. This is 32% higher than in BSRE4 and a remarkable 8.44-fold increase compared to BSR. Interestingly, it was noted that the growth and sucrose consumption rates of BSRE4/pMX45 were lower than those of BSRE4. (Fig. 5B, C, D)

### Optimization of the Fermentation Medium of BSRE4/pMX45

The sucrose consumption rate of BSRE4/pMX45, as illustrated in Fig. 5D, demonstrated a decline after 24 hours, specifically from 0.93 to 0.8 g/L/h. Furthermore, a noticeable rise in sugar accumulation occurred during the feeding process, as evidenced in Supplementary Fig. 4 Preliminary optimization efforts of the medium indicated that the levels of nitrogen sources, specifically corn steep powder, yeast extract, and (NH_4_)_2_SO_4_, had a substantial impact on riboflavin production and yield in BSRE4/pMX45. Consequently, we employed a Box-Behnken design and the response surface method (RSM) for further experimentation.”

To investigate the combined effects of these variables, we designed experiments with varying combinations using the Design Expert software. Table 2 summarizes the central composite experimental plan along with the experimental and predicted responses for each experiment. The corresponding equation could be written as:

$$\text{Y}=2998.39+157.89\text{A}+116.24\text{B}+27.39\text{C}$$

$$+240.47\text{A}\text{B}+9.65\text{A}\text{C}-102.79\text{B}\text{C}-104.84{\text{A}}^{2}$$

$$+115.53{\text{B}}^{2}-254.85{\text{C}}^{2}-77.31{\text{A}}^{2}\text{B}$$

$$-202.14{\text{A}}^{2}\text{C}-325.57\text{A}{\text{B}}^{2}$$

where R^2^ was 0.9711. Among the model terms, the linear coefficients for corn steep powder (*P* = 0.0275) and yeast extract (*P* = 0.0234) were more significant compared to other factors, indicating a substantial influence of their concentrations on riboflavin production. According to the equation, the optimized nitrogen sources for riboflavin production are as follows: corn steep powder 75 g/L, yeast extract 11.27 g/L, and (NH_4_)_2_SO_4_ 7.5 g/L, with a predicted production of 3513 mg/L. Under optimal conditions, we carried out shaking-flask cultivation, achieving a yield of 3477±231 mg/L, which closely matched the predicted values, indicating good agreement between experimental and theoretical outcomes.

**Table 2:** Central composite experimental plan and results

corn steep powder (g/L)	yeast extract (g/L)	(NH_4_)_2_SO_4_ (g/L)	riboflavin production (g/L)
30	12.5	20	2595
18.75	12.5	12.5	2882
18.75	5	5	2562
30	12.5	5	2925
7.5	5	12.5	3292
30	5	12.5	2476
18.75	20	20	2949
30	20	12.5	3134
18.75	20	5	3100
7.5	12.5	20	2260
7.5	12.5	5	2629
18.75	12.5	12.5	2920
18.75	5	20	3023
7.5	20	12.5	2989

## Discussion

In this study, we overexpressed the genes involved in downstream purine *de novo* synthesis, along with *ribA*, within a synthetic operon. The production of riboflavin exhibited a noteworthy increase when the synthetic operon was either integrated into the genome or introduced into BSR via a plasmid. Nevertheless, individual expression of the five genes revealed that only the expression of *ribA* resulted in augmented production compared to the parent strain. The combined expression of *ribA* with the other four genes, as well as four out of the five gene combinations, emphasized the profound influence of the *gmk* and *guaB*-*guaA* combination on riboflavin production. The optimization of fermentation media, utilizing the response RSM, unveiled a favorable effect of organic nitrogen sources on both riboflavin production and yield. It is worth mentioning that the optimal concentrations of corn steep powder and yeast extract were determined to be 4–6 times greater than their initial levels. These nitrogen source concentrations were notably higher than those commonly employed in the medium for the production of other chemicals by *B. subtilis*, as documented in previous studies [31–35].

This finding implies that a higher nitrogen input during the fermentation process is necessary to attain enhanced riboflavin production by the strain. Therefore, further research is needed to investigate the assimilation and utilization of nutrients in greater detail. Nitrogen sources are critical in supplying the essential precursors for amino acid and purine/pyrimidine biosynthesis in bacteria.

In the case of BSRE4/pMX45, the increased demand for nitrogen sources can be attributed to several factors. Firstly, the non-oxidative pentose phosphate pathway is impaired due to the presence of the *rpe* point mutation and *tkt* nonsense mutation. These mutations have the potential to disrupt the regular carbon and energy flux within the cell, resulting in an increased demand for nitrogen sources to offset the metabolic inefficiencies. Furthermore, a competition exists for the utilization of GTP between RNA synthesis and riboflavin biosynthesis in BSRE4/pMX45. This competition further intensifies the requirement for augmented nitrogen sources since GTP is a crucial element in both processes. Moreover, while the overexpression of genes is advantageous for boosting riboflavin production, it can impose a metabolic burden on the engineered strains. This burden can be alleviated by supplementing the culture medium with components that contain amino acids and nucleotides, such as corn steep powder and yeast extract, as previously described [36, 37]. These components supply vital precursors and cofactors necessary for diverse biosynthetic pathways, thereby enhancing the strain’s capacity to produce riboflavin efficiently.

Jiajia You and colleagues examined the impact of dissolved oxygen limitation on riboflavin production and downregulated the regulators *tnrA* and *glnR*, which are responsible for balancing intracellular nitrogen metabolism [38]. The low levels of dissolved oxygen in the flask environment may result in unfavorable conditions for riboflavin production owing to the inhibitory influence of *tnrA* and *glnR* on nitrogen source assimilation and utilization [39–41]. As a result, it was observed that medium optimization, specifically by elevating organic nitrogen source concentrations and reducing inorganic nitrogen source concentrations, led to the suppression of *tnrA* expression during cell cultivation [42]. The BSRE4/pMX45 strain was derived from the *B.subtilis* 168 background. In numerous references, it’s been noted that strains engineered from *B.subtilis* 168 tend to exhibit low biomass in fed-batch or batch fermentation processes [43–45]. As a result, flask fermentation is frequently employed for the evaluation of engineered riboflavin-producing strains. In fed-batch fermentation, the biomass of BSRE4/pMX45 remains low, exhibiting a measured OD_600_ value of 40. This low biomass does not pose a hindrance when producing primary metabolites like ribose and acetoin. Nonetheless, it is crucial to emphasize that riboflavin production is tightly associated with cell growth. Therefore, the riboflavin producing strains should undergo genetic mutations that enable continuous growth in the bioreactor, offering distinct advantages in this context.

By reducing the metabolic flux in the pyrimidine pathway, Xu et al. successfully increased riboflavin production to 7.01 g/L using 80 g/L of glucose, resulting in a yield of 0.089 g riboflavin/g glucose [46]. Zhang et al. also enhanced riboflavin production and yield through in vitro and in vivo modifications of the PP pathway, raising the yield from 0.065 g riboflavin/g glucose to 0.084 g riboflavin/g gluconate [47]. However, even compared with the industrial producer [48], the yield of inosine from engineered strains surpassed that of riboflavin. Takayuki Asahara et al. constructed a *B. subtilis* strain capable of inosine production through the introduction of just seven gene-targeted mutations [49]. This engineered strain achieved an impressive yield of 6 g/L inosine when supplied with 30 g/L of glucose, resulting in a remarkable yield of 1 g of inosine for every 5 g of glucose consumed. Notably, given that the yield of ribose/glucose was even higher [15], the purine *de novo* synthesis pathway has long been recognized as a bottleneck in riboflavin production. This leads us to hypothesize that the purine *de novo* biosynthesis pathway, especially downstream reactions involved in GTP synthesis, represents a significant limitation in terms of production and yield enhancement. In this study, we have partially validated this hypothesis and suggest that further experiments, such as the knockout of 5’-nucleotidases, warrant thorough investigation to enhance our understanding in this field. More importantly, the strategy of arranging previously dispersed genes into an artificial operon mimics nature’s deliberate biosynthetic processes that have occurred over the course of evolution. The implementation of artificial operons in metabolic engineering holds significant potential and provides a valuable framework for future research endeavors in microbial cell factories.

## Conclusions

In an effort to enhance riboflavin production in *B. subtilis*, a strategic focus was directed toward optimizing the downstream reactions associated with GTP *de novo* synthesis. In summation, these particular steps were identified as the predominant limiting factors for riboflavin production, particularly when an ample supply of pentose precursors was at hand. The chosen expression strategy demonstrated remarkable efficacy in mitigating these limitations. Additionally, it is of noteworthy significance that the engineered strain exhibited a pronounced dependency on organic nitrogen sources to attain higher production levels. This insight offers valuable contributions to the ongoing efforts in fine-tuning growth medium optimization for these strains.

## Material and method

### Strains and plasmids

The strains and plasmids used in this study are listed in Table 3. *B. subtilis* R (BSR) was derived from *B. subtilis* 168 by genome engineering in our previous work [14]. The genes cloned in all experiments described in the paper are from *B. subtilis* 168. BSR is a riboflavin production strain with deregulated *rib* operon and a mutant of *rpe* on the genome. *E. coli* DH5α was used as the host strain for genetic manipulation. Luria-Bertani medium was used for standard cultures of *B. subtilis* and *E. coli* unless indicated otherwise. For investigation of the riboflavin production and biomass, the culture was inoculated into a fermentation medium containing: 40 g/L sucrose, 15 g/L corn steep powder (Solarbio, Beijing, China), 7.5 g/L (NH_4_)_2_SO_4_, 5 g/L yeast extract (Solarbio, Beijing, China), 4 g/L MgSO_4_, 3 g/L K_2_HPO_4_, 1 g/L KH_2_PO_4_, and appropriate antibiotics. The optimization of the medium is based on this fermentation medium. A mid-copy number plasmid pHP13(spe) was constructed by replacing the original antibiotic-resistant gene with a spectinomycin-resistant gene [14, 50], which was used for the expression of all genes. pMX45 was used to overexpress the *rib* operon [49].

**Table 3:** Strains and plasmids used in this study

Strains or plasmids	Description of genotype	Source
Strains		
*B. subtilis* R (BSR)	*B.subtilis* 168, *araR*: para-*neo*, ribO*, *rib*+(*gsiB*), purA^*^, rpe^*^	[14]
BEX5	BSR/pEX5	This study
B13	BSR/pHP13(spe)	This study
BSRE1	BSR, △*amyE*::Pr-*ribA*-*ndk*-PvegI-*guaB*-*guaA*-*gmk*	This study
BSRE2	BSRE1, Δ*apt*	This study
BSRE3	BSRE2, *perR*^L71V^	This study
BSRE4	BSRE3, Δ*tkt*(tkt^0^)	This study
BSRE4/pMX45	BSRE4/pMX45	This study
BSRA	BSR/pEXRA	This study
BSGA	BSR/pEXGA	This study
BSGB	BSR/pEXGB	This study
BSGK	BSR/pEXGK	This study
BSNK	BSR/pEXNK	This study
BSAA	BSR/pEX2AA	This study
BSAB	BSR/pEX2AB	This study
BSAN	BSR/pEX2AN	This study
BSAG	BSR/pEX2AG	This study
BSRNBA	BSR/pEX4RNBA	This study
BSRBAG	BSR/pEX4RBAG	This study
BSRNBG	BSR/pEX4RNAG	This study
BSRNAG	BSR/pEX4RNAG	This study
BSNBAG	BSR/pEX4NBAG	This study
BSR1	BSR, Δ*apt*	[20]
BSR2	BSR, Δ*xpt*	[20]
BSR3	BSR, Δ*hprT*	[20]
BSR4	BSR, Δ*adeC*	[20]
BSR5	BSR, Δ*pucABCDE*	[20]
BSR6	BSR, Δ*yfkN*	[20]
BSR7	BSR, Δ*yunD*	[20]
BSR8	BSR, Δ*yktC*	[20]
BSR9	BSR, Δ*ycsE*	[20]
BSR10	BSR, Δ*deoD*	[20]
BSR11	BSR, Δ*pupG*	[20]
BSRPE1	BSR1/pEX5	This study
BSRPE2	BSR2/pEX5	This study
BSRPE3	BSR3/pEX5	This study
BSRPE4	BSR4/pEX5	This study
BSRPE5	BSR5/pEX5	This study
BSRPE6	BSR6/pEX5	This study
BSRPE7	BSR7/pEX5	This study
BSRPE8	BSR8/pEX5	This study
BSRPE9	BSR9/pEX5	This study
BSRPE10	BSR10/pEX5	This study
BSRPE11	BSR11/pEX5	This study
E.coli	Standard cloning strain	This study
Plasmids		
pHP13 (spc)	Spe^r^	[14]
pEX5	pHP13(spe)-Pr-*ribA*-*ndk*-PvegI-*guaB*-*guaA*-*gmk*, Spe^r^	This study
pEX4RNBA	pHP13(spe)-Pr-*ribA*-*ndk*-PvegI-*guaB*-*guaA*, Spe^r^	This study
pEX4RBAG	pHP13(spe)-Pr-*ribA-*PvegI-*guaB*-*guaA*-*gmk*, Spe^r^	This study
pEX4RNBG	pHP13(spe)-Pr-*ribA*-*ndk*-PvegI-*guaB*-*gmk*, Spe^r^	This study
pEX4RNAG	pHP13(spe)-Pr-*ribA*-*ndk*-PvegI-*guaA*-*gmk*, Spe^r^	This study
pEX4NBAG	pHP13(spe)-Pr-*ndk*-PvegI-*guaB*-*guaA*-*gmk*, Spe^r^	This study
pEXRA	pHP13(spe)-Pr-*ribA*, Spe^r^	This study
pEXGA	pHP13(spe)-Pr-*guaA*, Spe^r^	This study
pEXGB	pHP13(spe)-Pr-*guaB*, Spe^r^	This study
pEXGK	pHP13(spe)-Pr-*gmk*, Spe^r^	This study
pEXNK	pHP13(spe)-Pr-*ndk*, Spe^r^	This study
pEX2AA	pHP13(spe)-Pr-*ribA*-*guaA*, Spe^r^	This study
pEX2AB	pHP13(spe)-Pr-*guaA-guaB*, Spe^r^	This study
pEX2AN	pHP13(spe)-Pr-*guaA-ndk*, Spe^r^	This study
pEX2AG	pHP13(spe)-Pr-*guaA-gmk*, Spe^r^	This study
pMX45	Ery^r^, SM19035-derived plasmid, carrying a complete rib operon	[48]

### Genome manipulation and plasmid construction

*B. subtilis* transformation was performed according to a published protocol [51]. The genome manipulation method is based on the paraR-*neo*/*cat*-*araR* counter-selection system, the same as previous studies [52]. Taking BEX5 as an example: the synthetic operon, which cloned from pEX5 was integrated at the *aymE* locus of BSR to generate BSRE1. The primer pairs AmyE-ex5-UP1/AmyE-ex5-UP2 and AmyE-ex5-DN1/AmyE-ex5-DN2 were used to clone the upstream and downstream sequences flanking the *amyE* locus. The primer pair CR1/AmyE-ex5-CR2 was used to clone the *cat*-*araR* (CR) fragment, while AmyE-ex5-CR2 also carried a direct repeat (DR, 20 bp) sequence, the homologous DR sequence was also located downstream of the UP fragment. The primer pair AmyE-ex5-ex5-1/AmyE-ex5-ex5-2 was used to clone the synthetic operon from pEX5, which was introduced into the downstream of CR fragment by overlap Polymerase Chain Reaction (PCR). Other fragments above were also assembled by overlapped PCR, with the nucleotides at the upstream and *cat*-*araR* end. This fragment was used to transform BSR, after which Cm-resistant transformants were selected, and verified by PCR using the primer pair AmyE-ex5-UP1/AmyE-ex5-DN2. The PCR fragment was sequenced to prevent the mutations. The verified clone was incubated in LB broth for 8 h at 37℃ to induce intra-genomic recombination at the two homologous DR fragments, after which the culture was plated onto LB agar plates containing Nm. After incubation at 37◦C for 1 day, Nm-resistant colonies were selected and verified by PCR using the primer pair AmyE-ex5-UP1/AmyE-ex5-DN2. Standard protocol was used for the construction, purification, and analysis of plasmid DNA and other DNA fragments (See Table 4).

**Table 4:** Primers used in this study

Name	Sequence (5’–3’)
tkt-ko-UP1	AGCCAGCGGATATGAAATG
tkt-ko-UP2	TGAACTAATGGGTGCTTTAGTTGAAGAGTGCGAATGGTAGCAACTGAT
tkt-ko-CR2	CTGAGAAGCCGTATTCGTTAATGTGCGAATGGTAGCAACTGATTTCTTTTCAATTGTATCCATTATTCATTCAGTTTTCGTGCG
tkt-ko-DN1	ATTAACGAATACGGCTTCTCAG
tkt-ko-DN2	GCGGGTGATGAATGATTGC
apt-KO-UP1	GCAGAAATGAACATGCTGTCTCC
apt-KO-UP2	TGAACTAATGGGTGCTTTAGTTGAAGACCCTCTTTCGGGTA ATCCGGTAC
apt-KO-CR2	TGCCGTCAAGGTAAGAAAGCTCGATTAAGAAAGCGCCCTC TTTCGGGTAATCCGGTACAATTGTTACGTATTGTTTTATTC ATTCAGTTTTCGTGCG
apt-KO-DN1	CGCTTTCTTAATCGAGCTTTCTTACCTTG
apt-KO-DN2	GCTTATTTTGCAGCACCATTTTTCG
perR-KO-UP2	TGAACTAATGGGTGCTTTAGTTGAAGAGGCTTCTTTTAGTT CATGTGCA
perR-KO-CR2	ATGATTTTCTTTTTTCGAACACTCTTGGGGCTTCTTTTAGTT CATGTGCAGCCATCCGTCATGCACCTTTATTCATTCAGTTT TCGTGCG
perR-KO-DN1	CCAAGAGTGTTCGAAAAAAGAA
perR-KO-DN2	TTCGGCAAACGGAAATGTA
CR1	TCTTCAACTAAAGCACCCATTAGTTCAACAAACG
pHP13-2	AAGCCTGGGGTGCCTAATGAGT
ph13-pr	CTCACTCATTAGGCACCCCAGGCTTAATTGACGGCTCAGAGTATCATG
ribA-1	ATGTTTCATCCGATAGAAGAAGCACTGG
Pr-(ribA)-2	TAAAGCGTCCAGTGCTTCTTCTATCGGATGAAACATTTTGAATTCCTCCTTTTGTCCTTATTGGTTA
ex5-ribA-2	CCATCATTATGTATGTTAGAAATGAAGTAAATGACCTAGCTTGTTCAT
ex5-ndk-1	CATACATAATGATGGGGTGGAGAATATGATG
ex5-ndk-2	GAATATTGACTCTTGACGGTGAACTTGCTTAATAGATCCAGCCTGCCATAAGCT
ex5-PvegI-1	GCAAGTTCACCGTCAAGAGTC
ex5-PvegI-2	CGTTAAGCCTTCTTTTGAAAATTTACTTTCCCACATTGCATCCACCTCACTACATTTATTGTAC
ex5-guaB-1	ATGTGGGAAAGTAAATTTTCAAAAGAAGGC
ex5-guaB-2	CCTAATCTCCTCTAAGTTTATGAAATTGTATAGTTAGGTGATTCTTTTGTAATCTG
ex5-guaA-1	ACTTAGAGGAGATTAGGTGACAACCAT
ex5-guaA-2	CATGCGGCAATTACCCCTGCCCTTCATCTTATTCCCACTCAATCGTCGCAG
ex5-gmk-1	GATGAAGGGCAGGGGTAATTGC
ex5-gmk-2	CGACGGCCAGTGAATTTATTCAACCTCCAGCATTTTCTTATATCTT
pHP13-1	ATTCACTGGCCGTCGTTTTACAAC
ex4-guaA-3	CGACGGCCAGTGAATTTATTCCCACTCAATCGTCGCAG
ex4-guaB-3	CCCCTGCCCTTCATCTTATGAAATTGTATAGTTAGGTGATTCTTTTGTAATCTG
ex4-ribA-3	TGACGGTGAACTTGCTTAGAAATGAAGTAAATGACCTAGCTTGTTCAT
ex4-PvegI-3	TAATCTCCTCTAAGTTGCATCCACCTCACTACATTTATTGTAC
ex4-pr	CCATCATTATGTATGTTTGAATTCCTCCTTTTGTCCTTATTGGTTATTA
ex2-ribA-guaA	TAATCTCCTCTAAGTTTAGAAATGAAGTAAATGACCTAGCTTGTTCAT
ex2-ribA-gmk	CCCCTGCCCTTCATCTTAGAAATGAAGTAAATGACCTAGCTTGTTCAT
ex2-ribA-guaB	TTTACTTTCCCACATTTAGAAATGAAGTAAATGACCTAGCTTGTTCAT
ex2-guaB-3	AACGACGGCCAGTGAATTTATGAAATTGTATAGTTAGGTGATTCTTTTGTAATCTG
ex5-ribA-2	CCATCATTATGTATGTTAGAAATGAAGTAAATGACCTAGCTTGTTCAT
ex2-ndk-3	CGACGGCCAGTGAATTTAATAGATCCAGCCTGCCATAAGCT
Pr-ndk-oe-1	AAGGAGGAATTCAAACATACATAATGATGGGGTGGAGAATATGATG
ndk-oe-2	CGACGGCCAGTGAATTTAATAGATCCAGCCTGCCATAAGCT
ribA-002	CGACGGCCAGTGAATTTAGAAATGAAGTAAATGACCTAGCTTGTTCAT
guaB-1	AAGGAGGAATTCAAAATGTGGGAAAGTAAATTTTCAAAAGAAGGC
guaB-2	CGACGGCCAGTGAATTTATGAAATTGTATAGTTAGGTGATTCTTTTGTAATCTGTACG
Pr-2	TTTGAATTCCTCCTTTTGTCCTTATTGGTTA
guaA-1	AAGGAGGAATTCAAAATGACAAAGTTAGTGAATGAAATGATTCTTGTC
guaA-2	CGACGGCCAGTGAATTTATTCCCACTCAATCGTCGCAG
gmk-1	AAGGAGGAATTCAAAATGAAAGAAAGAGGGTTATTAATCGTTCTC
gmk-2	CGACGGCCAGTGAATTTATTCAACCTCCAGCATTTTCTTATATCTTGG
ndk-1	AAGGAGGAATTCAAAATGATGGAAAAGACTTTTATCATGGTG
ndk-2	CGACGGCCAGTGAATTTAATAGATCCAGCCTGCCATAAGCTG
AmyE-ex5-UP1	GGCGTGAATGGGAAAAATAAGAGAG
AmyE-ex5-UP2	TTTGTTGAACTAATGGGTGCTTTAGTTGAAGACAGTACCTAAGTAACGGTTGCC
CR1	TCTTCAACTAAAGCACCCATTAGTTC
AmyE-ex5-CR2	TGCTACTCCATGATACTCTGAGCCGTCAATTCAGTACCTAAGTAACGGTTGCCAATTTGATACGATGTCGGTTATTCATTCAGTTTTCGTGCGGAC
AmyE-ex5-ex5-1	AATTGACGGCTCAGAGTATCATGG
AmyE-ex5-ex5-2	AAAACTGTATTTCTCGGTCCTCGTTACACCTTATTCAACCTCCAGCATTTTCTTATATCTT
AmyE-ex5-DN1	GGTGTAACGAGGACCGAGAAATAC
AmyE-ex5-DN2	CTCGCAGAATCAAGTGTTGTG

All of the expression plasmids were constructed using Gibson assembly (Supplementary Fig. 5). Taking pEX5 as an example, the five genes: *ribA*, *guaB*, *guaA*, *gmk*, *ndk* was cloned from the genome of *B.subtilis* 168 by PCR using the primer pairs ribA-1/ex5-ribA-2, ex5-ndk-1/ex5-ndk-2, ex5-guaB-1/ex5-guaB-2, ex5-guaA-1/ex5-guaA-2, and ex5-gmk-1/ex5-gmk-2. The Pr and PvegI promoter were cloned from the genome of *B. subtilis* R and *B.subtilis* 168 with the primer pairs Pr-(ribA)-2/ph13-pr and ex5-PvegI-1/ex5-PvegI-2, respectively. The vector was amplified by PCR using primer pair pHP13-1/pHP13-2. The fragments of Pr, *ribA*, and *ndk* were fused into a longer fragment F1 with primer pair ph13-pr/ex5-ndk-2. And fragments of *guaB*, *guaA*, and *gmk* were fused into a longer fragment F2 with primer pair ex5-guaB-1/ex5-gmk-2. The F1, F2, and pHP13(spe) fragments were assembled using the Clon Expressmulti kit (Vazyme, Nanjing, China).

### Measurement of cell density and Riboflavin Titers

During fermentation, samples were taken at specific times to measure the cell growth and riboflavin production. The fermentation time was extended to 48–72 h until the production is not increasing. Cell growth was monitored by measuring the optical density (OD) at 600 nm. Determination and calculation of riboflavin concentration was performed as previous paper with some modifications [53]. Culture samples were diluted with 0.1 M NaOH to the linear range of the MAPADA V-1600 spectrophotometer (MAPADA, Shanghai, China). After dissolving for 20 min, 1 mL of the sample was centrifuged at 10,000 g for 2 min to remove the cells and other insoluble substances. Then the absorbance at 444 nm was immediately measured. The riboflavin concentration was calculated using a standard equation. All the fermentation experiments were performed in triplicates, and the reported results represented the average of three independent experiments.

### Optimization of Fermentation Medium compositions

To explore the interactions and determine the precise levels of media compositions, including yeast extract, corn steep powder, and (NH_4_)_2_SO_4_, significantly influencing riboflavin production, we employed a Box-Behnken design for 3 variables at three levels (+ 1, 0, and − 1). This optimization method aimed to maximize riboflavin production by BSRE4/pMX45, building upon the insights gained from one-factor-at-a-time experiments [54]. The statistical matrix encompassed 14 experimental runs to fit the response surface, as detailed in Table 3, presenting the variables, their values, and the experimental design.

### Electronic supplementary material

Below is the link to the electronic supplementary material.

Supplementary Material 1

## Data Availability

and supporting materials. All data generated and analyzed during this study are included in this manuscript and in its Supplemental Table.

## Abbreviations

Ru5P: Ribose-5-phosphate
R5P: Ribose-5-phosphate
PRPP: 5-phospho-α-D-ribosyl-1-pyrophosphate
IMP: inosine mono-phosphate
XMP: xanthosine monophosphate
GMP: guanosine mono-phosphate
GTP: guanosine tri-phosphate
AMP: adenosine mono-phosphate
DARPP: 2,5-diamino-6-ribosylamino-4(3 H)-pyrimidinone-5′-phosphate
ARPP: 5-amino-6-(5′-phosphoribosylamino)uracil
ArPP: 5-amino-6-(5′-phosphoribitylamino)uracil
ArP: 4-(1-D-ribitylamino)- 5-amino-2,6-dihydroxypyrimidine
DHPB: 3,4- dihydroxy-2-butanone 4-phosphate
DRL: 6,7- dimethyl-8-ribityl-lumazine
PCR: Polymerase chain reaction
DR: direct repeat
Nm: neomycin
Cm: chloramphenicol
Ery: erythromycin
Spe: erythromycin

## References

[1] Von MJ, Bourgonje AR, Klaassen MAY, Alkhalifah HAA, Sadaghian SM, Vich V, Gacesa R, Gabriëls RY, Steinert RE, Jansen BH, Bulthuis MLC, Visschedijk VDHM, Festen MC, Weersma EAM, de Vos RK, Van P, Faber GH, Harmsen KN, Dijkstra HJM. Riboflavin supplementation in patients with Crohn’s disease [the RISE-UP study]. J Crohns Colitis. 2020 Jun;14:595–607. 10.1093/ecco-jcc/jjz208 PMC7303596 31873717

[2] Suwannasom N, Kao I, Pruß A, Georgieva R, Bäumler H. Riboflavin: the health benefits of a forgotten natural vitamin. Int J Mol Sci. 2020 Jan;21:950. 10.3390/ijms21030950 PMC7037471 32023913

[3] Bampidis V, Azimonti G, Bastos ML, Christensen H, Dusemund B, Fašmon Durjava M, Kouba M, López-Alonso M, López Puente S, Marcon F, Mayo B, Pechová A, Petkova M, Ramos F, Sanz Y, Villa RE, Woutersen R, Anguita M, Galobart J, Manini P, Pettenati E, Pizzo F. Safety and efficacy of a feed additive consisting of riboflavin-5’-phosphate ester monosodium salt (vitamin B2) (from riboflavin 98%, produced by *Bacillus subtilis* KCCM 10445) for all animal species (Hubei Guangji Pharmaceutical Co. Ltd). EFSA J. 2022 Nov;20:11.

[4] Abbas CA, Sibirny AA (2011). Genetic control of biosynthesis and transport of riboflavin and flavin nucleotides and construction of robust biotechnological producers. Microbiol Mol Biol Rev.

[5] Schwechheimer SK, Park EY, Revuelta JL, Becker J, Wittmann C (2016). Biotechnology of Riboflavin. Appl Microbiol Biotechnol.

[6] Balasubramaniam S, Christodoulou J, Rahman S (2019). Disorders of Riboflavin metabolism. J Inherit Metab Dis.

[7] Kil YV, Mironov VN, Gorishin IYu, Kreneva RA, Perumov DA (1992). Riboflavin operon of *Bacillus subtilis*: unusual symmetric arrangement of the regulatory region. Mol Gen Genet.

[8] Worst DJ, Gerrits MM, Vandenbroucke-Grauls CM, Kusters JG (1998). *Helicobacter pylori* ribBA-mediated riboflavin production is involved in iron acquisition. J Bacteriol.

[9] Sibirnyĭ AA, Shavlovskiĭ GM, Ksheminskaia GP (1978). Effect of glucose and its derivatives on systems of riboflavin uptake and excretion in the yeast *Pichia guilliermondii*. Biokhimiia.

[10] Hu YG, Yang MY, Liu YM, Zhou XT. Method for increasing yield of *Bacillus subtilis* vitamin B2. CN109134468A 2019.

[11] Christopher MF, Sébastien EP, Zoltán P, Michael PA. Improve production of Riboflavin. WO2020099303; 2020.

[12] Zhang GY, Chen Y. Vitamin B2 high-yield bacterial strain and preparation method thereof. CN107475142A; 2017.

[13] Riboflavin market - growth. trends, Covid-19 impact, and forecasts (2022–2027).https://www.mordorintelligence.com/industry-reports/riboflavin-market. Accessed 25 May 2022.

[14] Yang B, Sun Y, Fu S, Xia M, Su Y, Liu C, Zhang C, Zhang D (2021). Improving the production of riboflavin by introducing a mutant ribulose 5-phosphate 3-epimerase gene in *Bacillus subtilis*. Front Bioeng Biotechnol.

[15] De Wulf P, Vandamme EJ (1997). Production of D-ribose by fermentation. Appl Microbiol Biotechnol.

[16] Saxild HH, Brunstedt K, Nielsen KI, Jarmer H, Nygaard P. Definition of the Bacillus subtilis PurR operator using genetic and bioinformatic tools and expansion of the PurR regulon with glyA, guaC, pbuG, xpt-pbuX, yqhz-fold, and pbuO. J Bacteriol. 2001;183:6175–83. 10.1128/JB.183.21.6175-6183.2001 PMC100094 11591660

[17] Boumezbeur AH, Bruer M, Stoecklin G, Mack M. Rational engineering of transcriptional riboswitches leads to enhanced metabolite levels in *Bacillus subtilis*. Metab Eng. 2020;61:58–68. 10.1016/j.ymben.2020.05.002 32413407

[18] Smith JL, Zaluzec EJ, Wery J-P, Niu L, Switzer RL, Zalkin H, Satow Y. Structure of the allosteric regulatory enzyme of purine biosynthesis. Science. 1994;264:1427–33. 10.1126/science.8197456 8197456

[19] Shi T, Wang Y, Wang Z, Wang G, Liu D, Fu J, Chen T, Zhao X. Deregulation of purine pathway in *Bacillus subtilis* and its use in riboflavin biosynthesis. Microb Cell Fact. 2014;13:101. 10.1186/s12934-014-0101-8 PMC4223553 25023436

[20] Sun Y, Liu C, Tang W, Zhang D (2020). Manipulation of purine metabolic networks for riboflavin production in *Bacillus subtilis*. ACS Omega.

[21] Kunst F, Ogasawara N, Moszer I, Albertini AM, Alloni G, Azevedo V, Bertero MG, Bessières P, Bolotin A, Borchert S, Borriss R, Boursier L, Brans A, Braun M, Brignell SC, Bron S, Brouillet S, Bruschi CV, Caldwell B, Capuano V, Carter NM, Choi SK, Cordani JJ, Connerton IF, Cummings NJ, Daniel RA, Denziot F, Devine KM, Düsterhöft A, Ehrlich SD, Emmerson PT, Entian KD, Errington J, Fabret C, Ferrari E, Foulger D, Fritz C, Fujita M, Fujita Y, Fuma S, Galizzi A, Galleron N, Ghim SY, Glaser P, Goffeau A, Golightly EJ, Grandi G, Guiseppi G, Guy BJ, Haga K, Haiech J, Harwood CR, Hènaut A, Hilbert H, Holsappel S, Hosono S, Hullo MF, Itaya M, Jones L, Joris B, Karamata D, Kasahara Y, Klaerr-Blanchard M, Klein C, Kobayashi Y, Koetter P, Koningstein G, Krogh S, Kumano M, Kurita K, Lapidus A, Lardinois S, Lauber J, Lazarevic V, Lee SM, Levine A, Liu H, Masuda S, Mauël C, Médigue C, Medina N, Mellado RP, Mizuno M, Moestl D, Nakai S, Noback M, Noone D, O’Reilly M, Ogawa K, Ogiwara A, Oudega B, Park SH, Parro V, Pohl TM, Portelle D, Porwollik S, Prescott AM, Presecan E, Pujic P, Purnelle B, Rapoport G, Rey M, Reynolds S, Rieger M, Rivolta C, Rocha E, Roche B, Rose M, Sadaie Y, Sato T, Scanlan E, Schleich S, Schroeter R, Scoffone F, Sekiguchi J, Sekowska A, Seror SJ, Serror P, Shin BS, Soldo B, Sorokin A, Tacconi E, Takagi T, Takahashi H, Takemaru K, Takeuchi M, Tamakoshi A, Tanaka T, Terpstra P, Togoni A, Tosato V, Uchiyama S, Vandebol M, Vannier F, Vassarotti A, Viari A, Wambutt R, Wedler H, Weitzenegger T, Winters P, Wipat A, Yamamoto H, Yamane K, Yasumoto K, Yata K, Yoshida K, Yoshikawa HF, Zumstein E, Yoshikawa H, Danchin A. The complete genome sequence of the gram-positive bacterium *Bacillus subtilis*. *Nature*. 1997, 390: 249 – 56. 10.1038/36786 9384377

[22] Karp PD, Billington R, Caspi R, Fulcher CA, Latendresse M, Kothari A, Keseler IM, Krummenacker M, Midford PE, Ong Q, Ong WK, Paley SM, Subhraveti P. The BioCyc collection of microbial genomes and metabolic pathways. Brief Bioinform 2019. 10.1093/bib/bbx085 PMC6781571 29447345

[23] Terakawa A, Natsume A, Okada A, Nishihata S, Kuse J, Tanaka K, Takenaka S, Ishikawa S, Yoshida KI (2016). *Bacillus subtilis* 5’-nucleotidases with various functions and substrate specificities. BMC Microbiol.

[24] Humbelin M, Griesser V, Keller T (1999). GTP cyclohydrolase II and 3,4-dihydroxy-2-butanone 4-phosphate synthase are rate-limiting enzymes in riboflavin synthesis of an industrial *Bacillus subtilis* strain used for riboflavin production. J Ind Microbiol Biotechnol.

[25] Xia MM, Liu L, Ban R (2014). The genetic modification effect of *rib* operon and *ribC* gene in *Bacillus subtilis*. Microbiol China.

[26] Willenbacher J, Mohr T, Henkel M, Gebhard S, Mascher T, Syldatk C, Hausmann R (2016). Substitution of the native *srfA* promoter by constitutive Pveg in two *B. subtilis* strains and evaluation of the effect on surfactin production. J Biotechnol.

[27] Peter H, Sierd B, Gerard V (1987). The effect of restriction on shotgun cloning and plasmid stability in *Bacillus subtilis* Marburg. Mol Gen Genet.

[28] Liu S, Hu W, Wang Z, Chen T (2021). Rational Engineering of *Escherichia col*i for High-Level production of Riboflavin. J Agric Food Chem.

[29] Rinas U, Hellmuth K, Kang R, Seeger A, Schlieker H (1995). Entry of Escherichia coli into stationary phase is indicated by endogenous and exogenous accumulation of nucleobases. Appl Environ Microbiol.

[30] Grifantini R, Toukoki C, Colaprico A, Gryllos I (2011). Peroxide stimulon and role of PerR in group a Streptococcus. J Bacteriol.

[31] Yang S, Cao Y, Sun L, Li C, Lin X, Cai Z, Zhang G, Song H (2019). Modular pathway engineering of *Bacillus subtilis*to promote *de novo* biosynthesis of menaquinone-7. ACS Synth Biol.

[32] Janek T, Gudiña EJ, Połomska X, Biniarz P, Jama D, Rodrigues LR, Rymowicz W, Lazar Z (2021). Sustainable surfactin production by *Bacillus subtilis* using crude glycerol from different wastes. Molecules.

[33] Wu L, Li Z, Ye Q (2009). Enhanced D-ribose biosynthesis in batch culture of a transketolase-deficient *Bacillus subtilis* strain by citrate. J Ind Microbiol Biotechnol.

[34] Mohamed H, Awad MF, Shah AM, Sadaqat B, Nazir Y, Naz T, Yang W, Song Y (2022). Coculturing of mucor plumbeus and *Bacillus subtilis* bacterium as an efficient fermentation strategy to enhance fungal lipid and gamma-linolenic acid (GLA) production. Sci Rep.

[35] Zhang K, Tan R, Yao D, Su L, Xia Y, Wu J (2021). Enhanced production of soluble pyrococcus furiosus α-Amylase in *Bacillus subtilis* through chaperone co-expression, heat treatment and fermentation optimization. J Microbiol Biotechnol.

[36] Lin W, Li L, Jing Y, Liu XL (2018). Study on compositions and antioxidant activity of corn steep liquor. Food Ind.

[37] Chen J, Wang Z (2003). The reason for the low content of flavor enhancer (5’-GMP) in the yeast extract RNA degradation properties in yeast autolysis. Shi Pin Ke Xue.

[38] You J, Yang C, Pan X, Hu M, Du Y, Osire T, Yang T, Rao Z (2021). Metabolic engineering of *Bacillus subtilis* for enhancing riboflavin production by alleviating dissolved oxygen limitation. Bioresour Technol.

[39] Wray LV, Zalieckas JM, Fisher SH (2001). *Bacillus subtilis* glutamine synthetase controls gene expression through a protein–protein interaction with transcription factor TnrA. Cell.

[40] Yoshida K, Yamaguchi H, Kinehara M, Ohki YH, Nakaura Y, Fujita Y (2003). Identification of additional TnrA-regulated genes of *Bacillus subtilis* associated with a TnrA box. Mol Microbiol.

[41] Travis BA, Peck JV, Salinas R, Dopkins B, Lent N, Nguyen VD, Borgnia MJ, Brennan RG, Schumacher MA (2022). Molecular dissection of the glutamine synthetase-GlnR nitrogen regulatory circuitry in Gram-positive bacteria. Nat Commun.

[42] Belitsky BR, Wray LV, Fisher SH, Bohannon DE, Sonenshein AL (2000). Role of TnrA in nitrogen source-dependent repression of *Bacillus subtilis* glutamate synthase gene expression. J Bacteriol.

[43] Jin P, Liang Z, Li H, Chen C, Xue Y, Du Q (2021). Biosynthesis of low-molecular-weight mannan using metabolically engineered *Bacillus subtilis* 168. Carbohydr Polym.

[44] Zhang L, Wang H, Zhou Z, Du G, Chen J, Kang Z (2017). Optimization of heparosan synthetic pathway in *Bacillus subtilis* 168. Sheng Wu Gong Cheng Xue Bao.

[45] Park YC, Choi JH, Bennett GN, Seo JH (2006). Characterization of D-ribose biosynthesis in *Bacillus subtilis* JY200 deficient in transketolase gene. J Biotechnol.

[46] Xu J, Wang C, Ban R (2022). Improving riboflavin production by modifying related metabolic pathways in *Bacillus subtilis*. Lett Appl Microbiol.

[47] Zhang M, Zhao X, Chen X, Li M, Wang X (2021). Enhancement of riboflavin production in *Bacillus subtili*s via in vitro and in vivo metabolic engineering of pentose phosphate pathway. Biotechnol Lett.

[48] Zhdanov VG, Stepanov AI. Riboflavin preparation. Patent FR2546907A1 1984.

[49] Asahara T, Mori Y, Zakataeva NP, Livshits VA, Yoshida K, Matsuno K (2010). Accumulation of gene-targeted *Bacillus subtilis* mutations that enhance fermentative inosine production. Appl Microbiol Biotechnol.

[50] Wang G, Shi T, Chen T, Wang X, Wang Y, Liu D, Guo J, Fu J, Feng L, Wang Z, Zhao X (2018). Integrated whole-genome and transcriptome sequence analysis reveals the genetic characteristics of a riboflavin-overproducing *Bacillus subtilis*. Metab Eng.

[51] Anagnostopoulos C, Spizizen J (1961). Requirements for transformation in *Bacillus subtilis*. J Bacteriol.

[52] Liu S, Endo K, Ara K, Ozaki K, Ogasawara N (2008). Introduction of marker-free deletions in *Bacillus subtilis* using the AraR repressor and the ara promoter. Microbiology.

[53] Himanshi S, Shraddha P, Bhavik P, Nitin S, Nurudin PJ, Digbijay BK (2012). Development and validation of uv-visible spectrometric method for estimation of water soluble vitamin riboflavin. Int J Pharm Sci Res.

[54] Tahereh K, Mohammad JC, OmLeila N, Hamid G (2011). Application of Box–Behnken design in the optimization of catalytic behavior of a new mixed chelate of copper (ІІ) complex in chemiluminescence reaction of luminol. J Lumin.

