# Supplementary material for: De novo engineering riboflavin production Bacillus subtilis by overexpressing the downstream genes in the purine biosynthesis pathway
Source: Microb Cell Fact. 2024 May 31;23:159. doi: 10.1186/s12934-024-02426-w (PMC11141002; doi:10.1186/s12934-024-02426-w)
Supplement: Supplementary file 1 — Supplementary Material 1 [file 12934_2024_2426_MOESM1_ESM.docx]

**Supplementary** **Fig. 1** The map of plasmid Pex5 with pHP13(spe) [14, 26] as the backbone. The arrangement of the promoters and genes is as follows: Pr *ribA*, *ndk*, PvegI, *guaB*, *guaA*, *gmk*. The synthetic operon is ligated to backbone plasmid pHP13(spe) by Gibson assembly to generate pEX5.

**
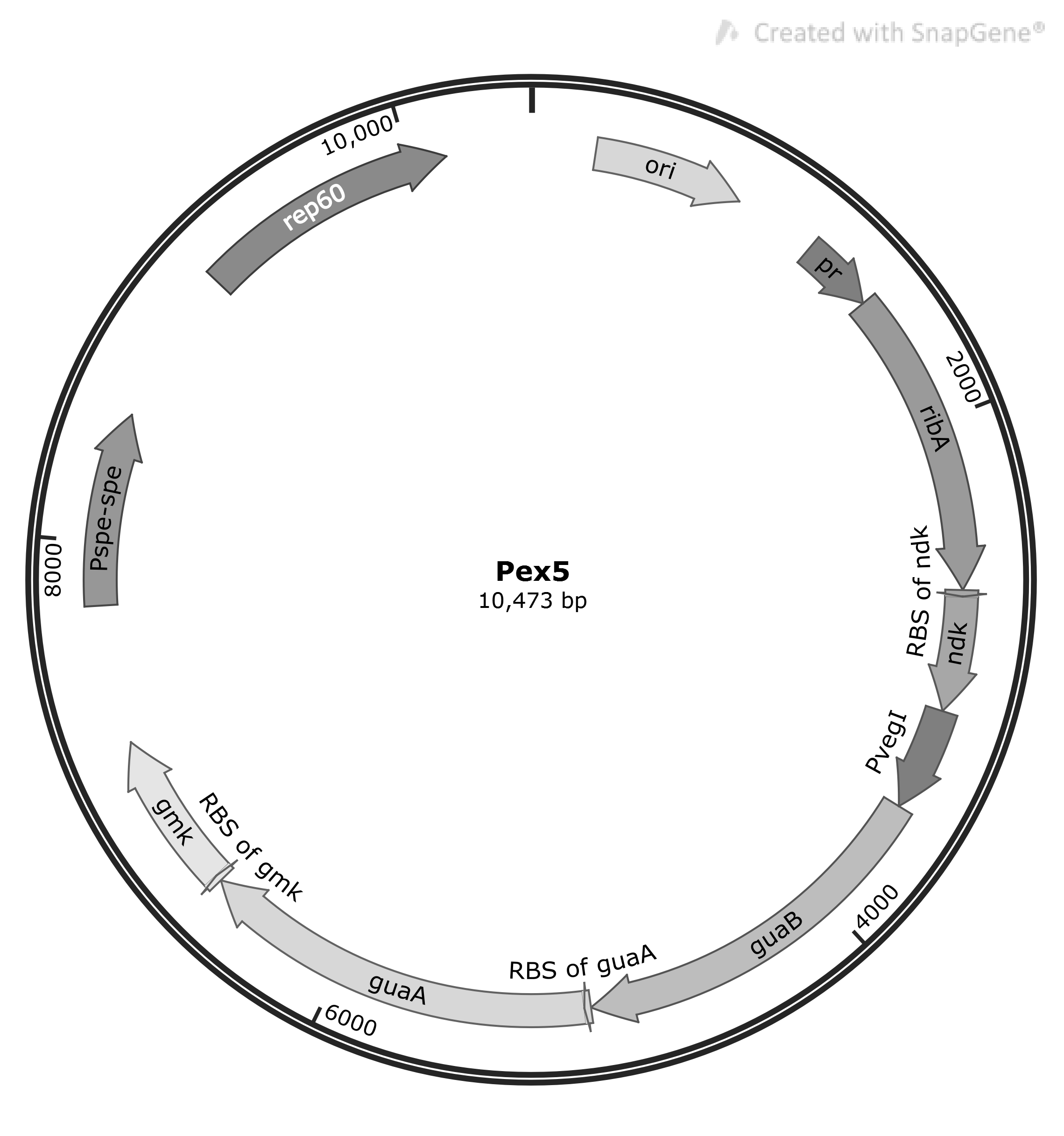
**

**
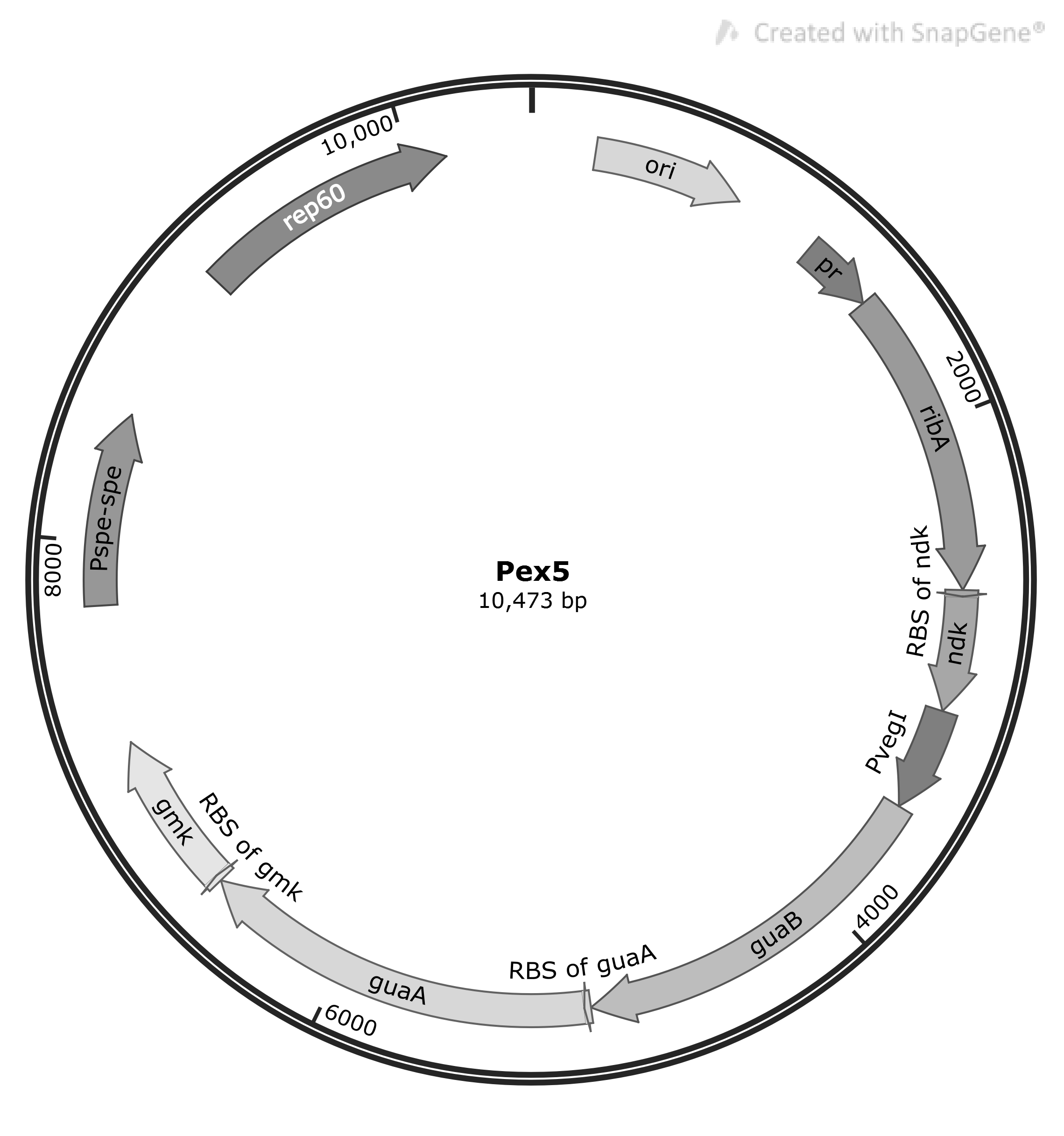
Supplementary** **Fig. 2** The biomass of strains individually overexpressing *guaB*, *guaA*, *gmk*, *ndk*, and *ribA* in the context of the BSR background.


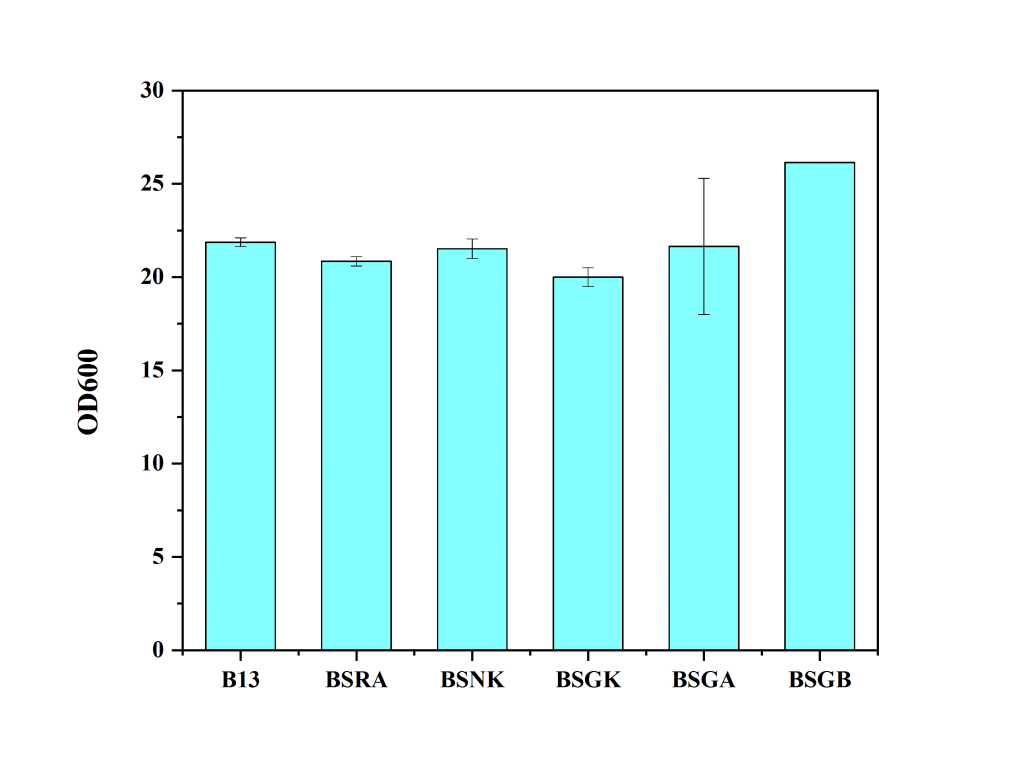


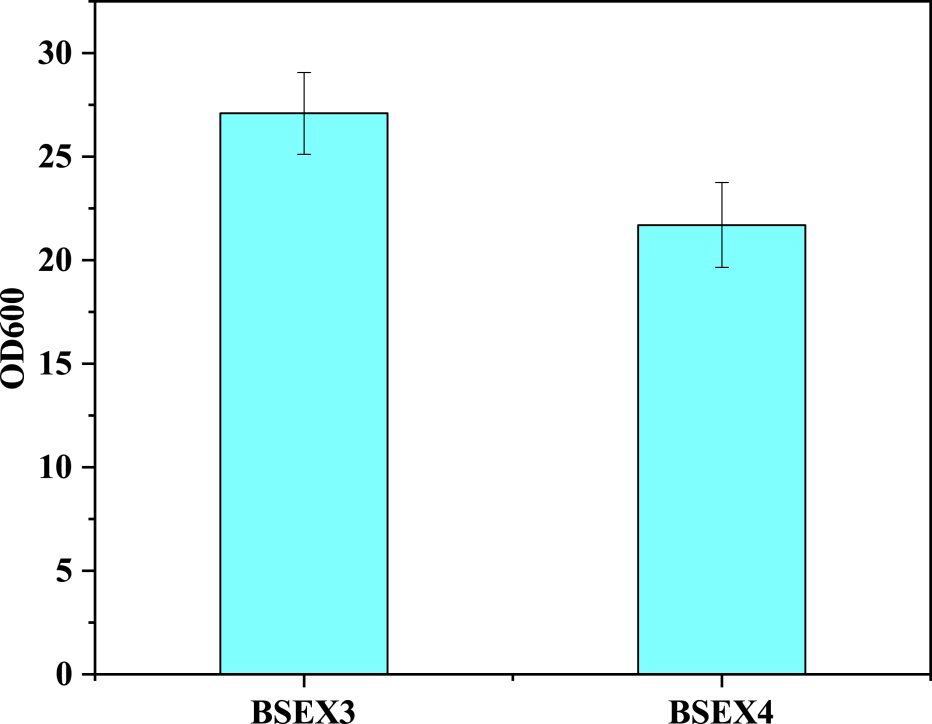


**Supplementary** **Fig. 3** The biomass of BSEX3 and BSEX4. Transketolase was knocked out on the background of parent strain (BSRE3), which contain the ribulose-5-phosphate 3-epimerase mutation.


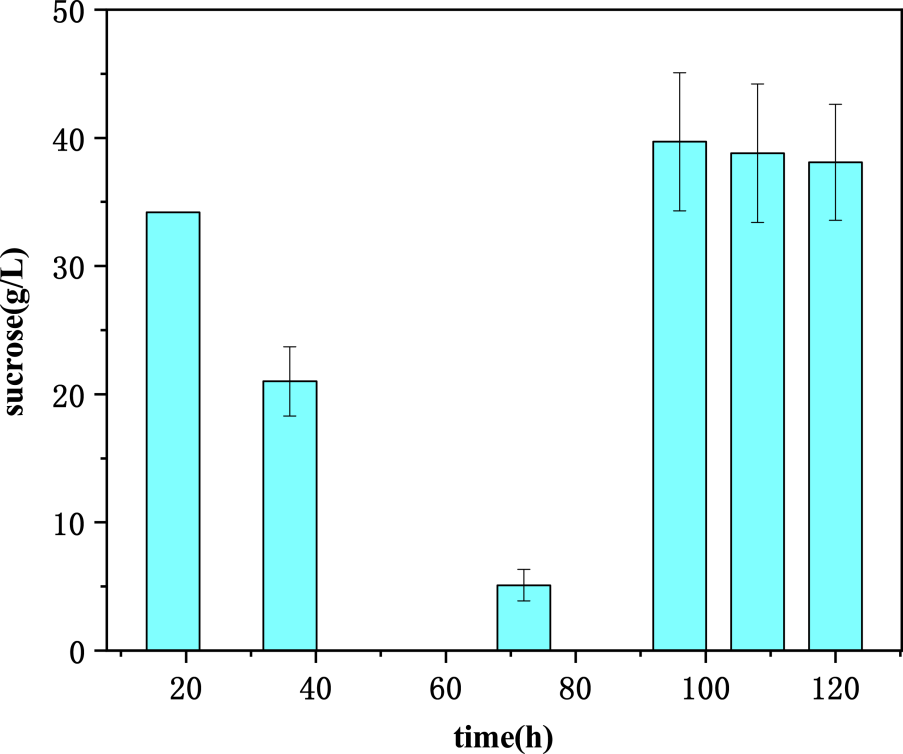


**Supplementary** **Fig. 4** The sucrose concentration of BSEX4/pMX45 in the flask fermentation. Feeding process was conduct after measurement of the sucrose concentration in the medium at 72 h.


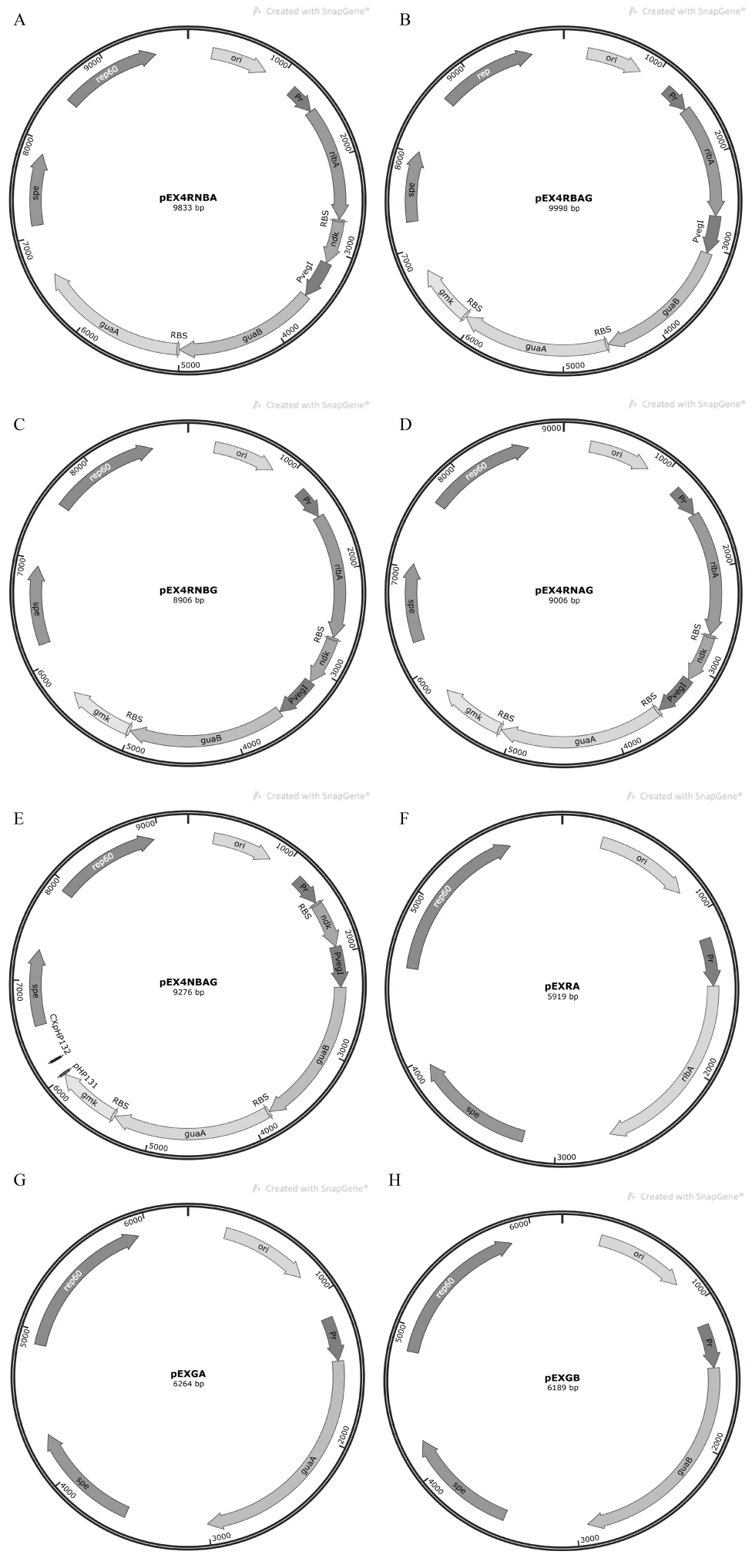


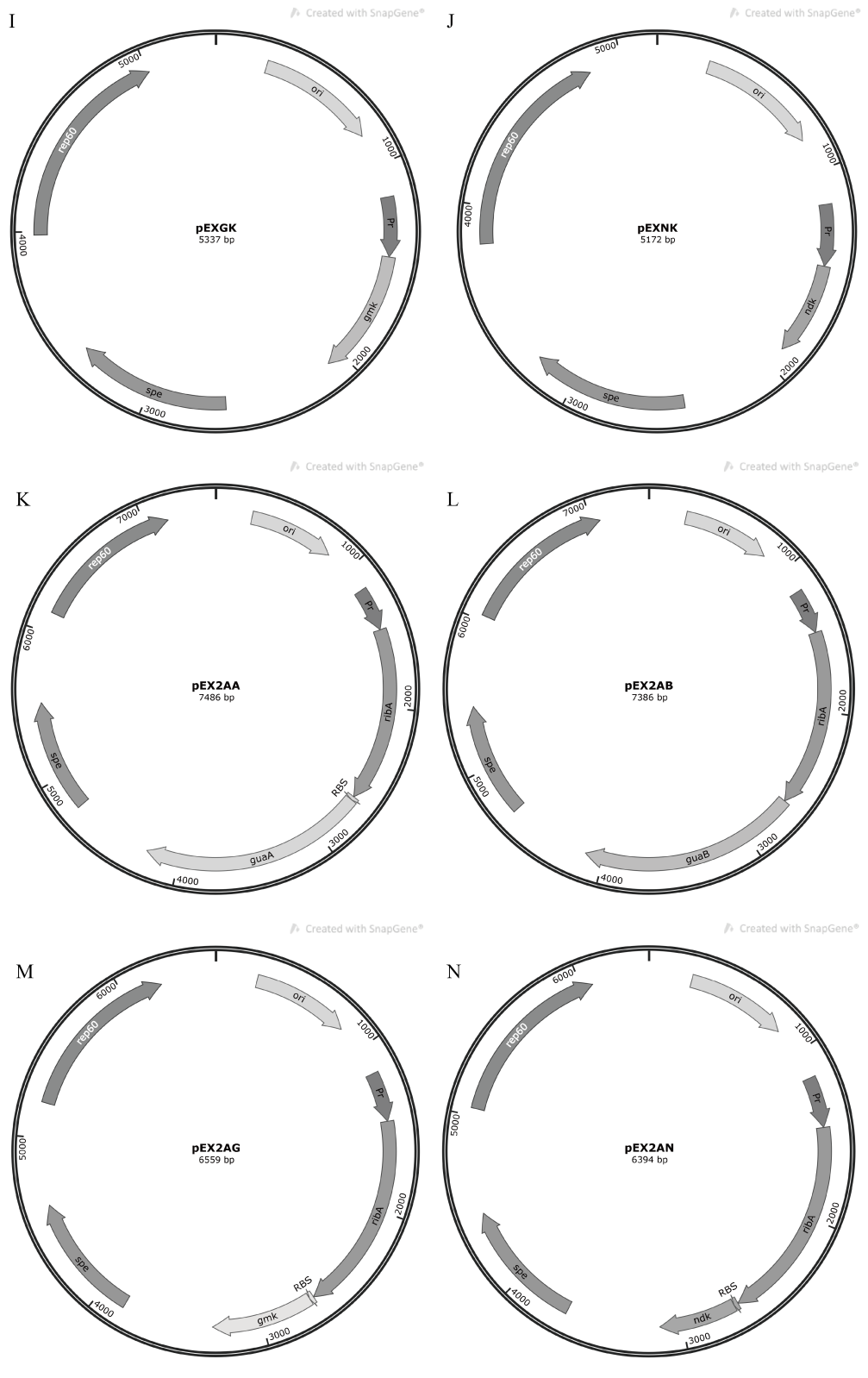


**Supplementary** **Fig. 5** Organization of Recombinant Plasmids. A-N correspond to the organization of recombinant plasmids, including pEX4RNBA, pEX4RBAG, pEX4RNBG, pEX4RNAG, pEX4NBAG, pEXRA, pEXGA, pEXGB, pEXGK, pEXNK, pEX2AA, pEX2AB, pEX2AN and pEX2AG as detailed in Table 3.

**
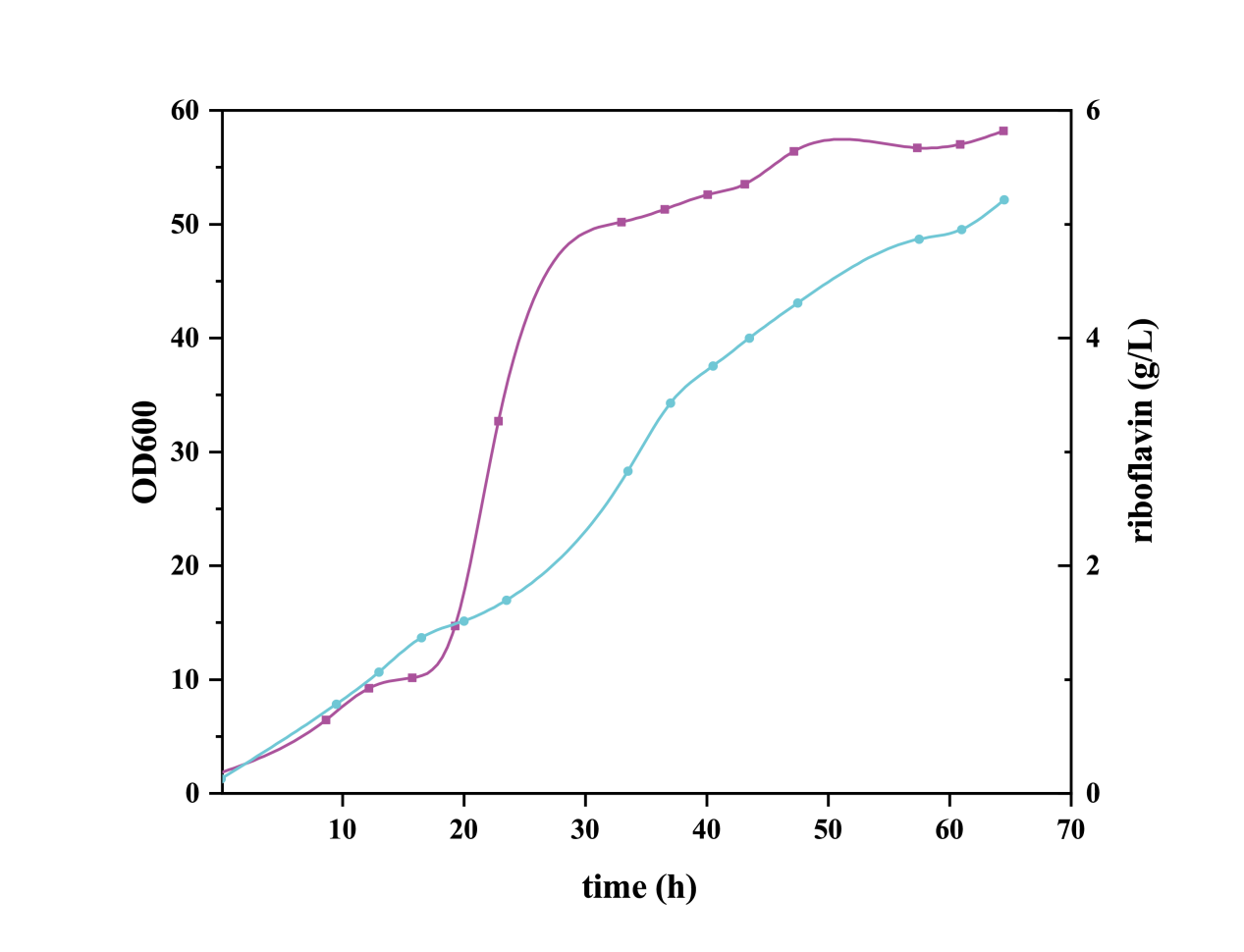
**
